# Bioactive Hemp Clothing Modified with Cannabidiol (CBD) *Cannabis sativa* L. Extract

**DOI:** 10.3390/ma14206031

**Published:** 2021-10-13

**Authors:** Malgorzata Zimniewska, Mariola Pawlaczyk, Barbara Romanowska, Agnieszka Gryszczyńska, Edyta Kwiatkowska, Patrycja Przybylska

**Affiliations:** 1 Natural Fibers and Medicinal Plants National Research Institute, Wojska Polskiego 71B, 60-630 Poznan, Poland; barbara.romanowska@iwnirz.pl (B.R.); agnieszka.gryszczynska@iwnirz.pl (A.G.); edyta.kwiatkowska@iwnirz.pl (E.K.); patrycja.przybylska@iwnirz.pl (P.P.); 2 Department and Division of Practical Cosmetology and Skin Diseases Prophylaxis, Poznan University of Medicinal Sciences, Mazowiecka 33, 60-623 Poznan, Poland; mariolapawlaczyk@ump.edu.pl

**Keywords:** hemp clothing, phenolic acid, cannabidiol (CBD), bioactivity, skincare

## Abstract

Hemp fiber variety, Bialobrzeskie, contains phenolic acids in its chemical composition giving it inherent antioxidant and antibacterial activity. The use of this raw material in fabric manufacture allows the creation of functional clothing with a positive effect on human skin. The aim of the study was to develop biologically active functional clothing made of pure industrial hemp raw materials, where cannabidiol (CBD) extract applied on the fabric surface strengthened the fiber bioactivity. The design of the clothing technology was focused on keeping the hemp inherent properties on a steady level and avoiding the use of chemicals in each stage of the value chain from plant cultivation up to garment manufacture. The research covered the evaluation of phenolic acids content and The Ferric Ion Reducing Antioxidant Power FRAP antioxidant activity of the hemp fabric. The hemp fabric enriched with CBD was used for clothing preparation. The human trials covered wearing of the clothing by 15 volunteers for six weeks and evaluation of hemp garment effect on human skin. The skin parameters were tested twice, before and after six weeks of clothing wearing, according to the own methodology that included measurements of skin biophysical properties including tests of skin moisture, transepidermal water loss, and sebum. Also, the effect of the active substances present on the fabrics on the in vitro culture of human keratinocytes was evaluated. Results of the research proved, that the wearing of developed functional hemp clothing with CBD extracts applied on the fabric surface was safe and caused improvement of skin condition, which can have an influence on slowing down of skin aging. The invention covering the pure hemp functional clothing with hybrid bioactivity resulting from the joined activity of fiber and cannabidiol was applied for a patent, Patent Application No: P.438388, 2021.

## 1. Introduction

**Figure 1:**
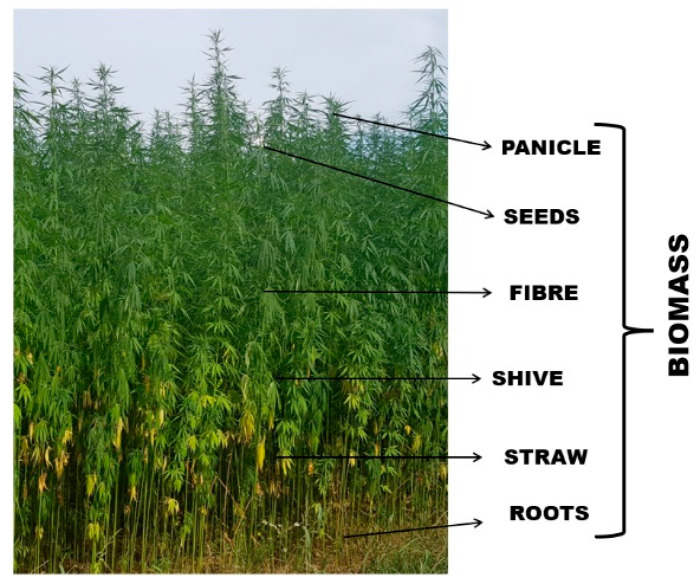
The hemp plant, variety Bialobrzeskie.

**Figure 2:**
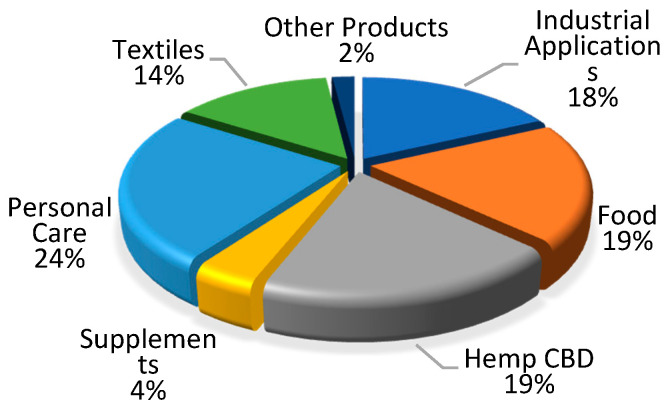
Hemp products sales by category, 2015, based on HIA, “2015 Annual Retail Sales for Hemp Products Estimated at 573 million of USD”, 9 May 2016.

*Cannabis sativa* L. is a plant highly appreciated due to its strong ecological features and potential for multidirectional application. The importance of hemp has increased especially nowadays where climate change and environmental degradation are serious problems for our globe. The European Green Deal launched as Europe’s growth strategy aims to make the economy more sustainable, resource-efficient, and competitive, focusing on ensuring: no net emissions of greenhouse gases by 2050, economic growth decoupled from resource use, no person and no place left behind [1]. Hemp fits into the European Green Deal priority particularly in terms of striving to climatic neutrality in 2050. Hemp cultivation and processing cover low-carbon solution idea, 1 ha of one season of hemp growing can absorb about 10 t of CO_2_ from the atmosphere, depending on plant variety. Hemp is resistant to drought, its growth does not need watering or too many pesticides, as opposed to cotton. Hemp is a model example of cascading wasteless use of biomass, which is presented in a simplified way in Figure 1. Each part of the plant is raw material suitable to use in different sectors of the economy bringing benefits to farmers and industries. The hemp value chain covers wasteless processing of biomass to produce biodegradable and recyclable products. The diversity of hemp products and their quantitative share in the market is shown in Figure 2. 

**Figure 3:**
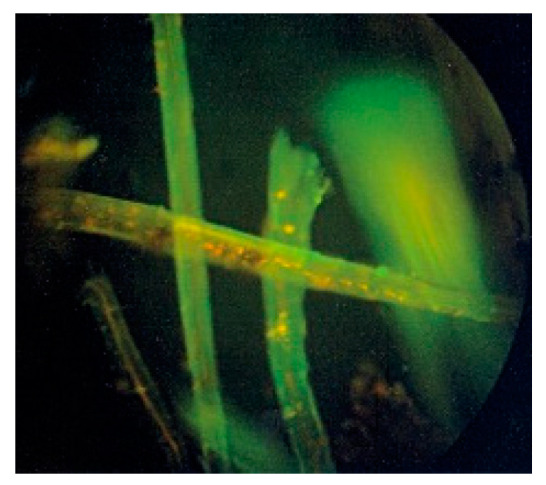
Dew retted hemp fibers activity towards *Staphylococcus aureus* bacteria, the killed bacterias take the form of orange particles. The strains of the clinical *Staphylococcus aureus* bacteria were isolated from ill people after 90 min of incubation.

Currently, the huge interest in hemp is also addressed to textiles. Hemp fiber was traditionally used for technical textiles, mainly for ropes due to its high mechanical properties. The effort of researchers and stakeholders are focused on the development of high quality fiber for textile purpose especially clothing for which the inherent bioactive properties of hemp fibers is added value. Hemp fibers are more difficult to processing in comparison to flax, they are more stiff and coarse. Raw hemp fibers show antibacterial and antioxidant properties resulted from fiber chemical composition, they contain phenolic acids known as natural antioxidants [2]. The antibacterial activity of raw hemp fibers is illustrated in Figure 3, where strains of the bacteria *Staphylococcus aureus* are located directly on the surface of pure hemp fiber. 

However, hemp technological processing can cause the removal of some amount of phenolic acids from the structure of the fiber resulting in a reduction of their bioactivity. Especially the retting process of hemp straw has a significant effect on fiber functionality. The earlier study proved that fibers extracted from the straw with the use of dew retting show the highest bioactivity in comparison to water retted fibers [2]. Hemp fiber bioactivity was found also in the case of use of mechanical extraction from the straw, e.g., decortication without retting process, but such fibers are coarse and contain a high amount of impurities that make them not suitable for the textile purpose. 

The challenge of the development of hemp clothing technological chain allowing to keep fiber properties on the acceptable level was taken up in the current study. Additionally CBD extracted from industrial hemp plant panicles was applied on the fabric surface to strengthen the antioxidant and antibacterial activity of the clothing. 

**Figure 4:**
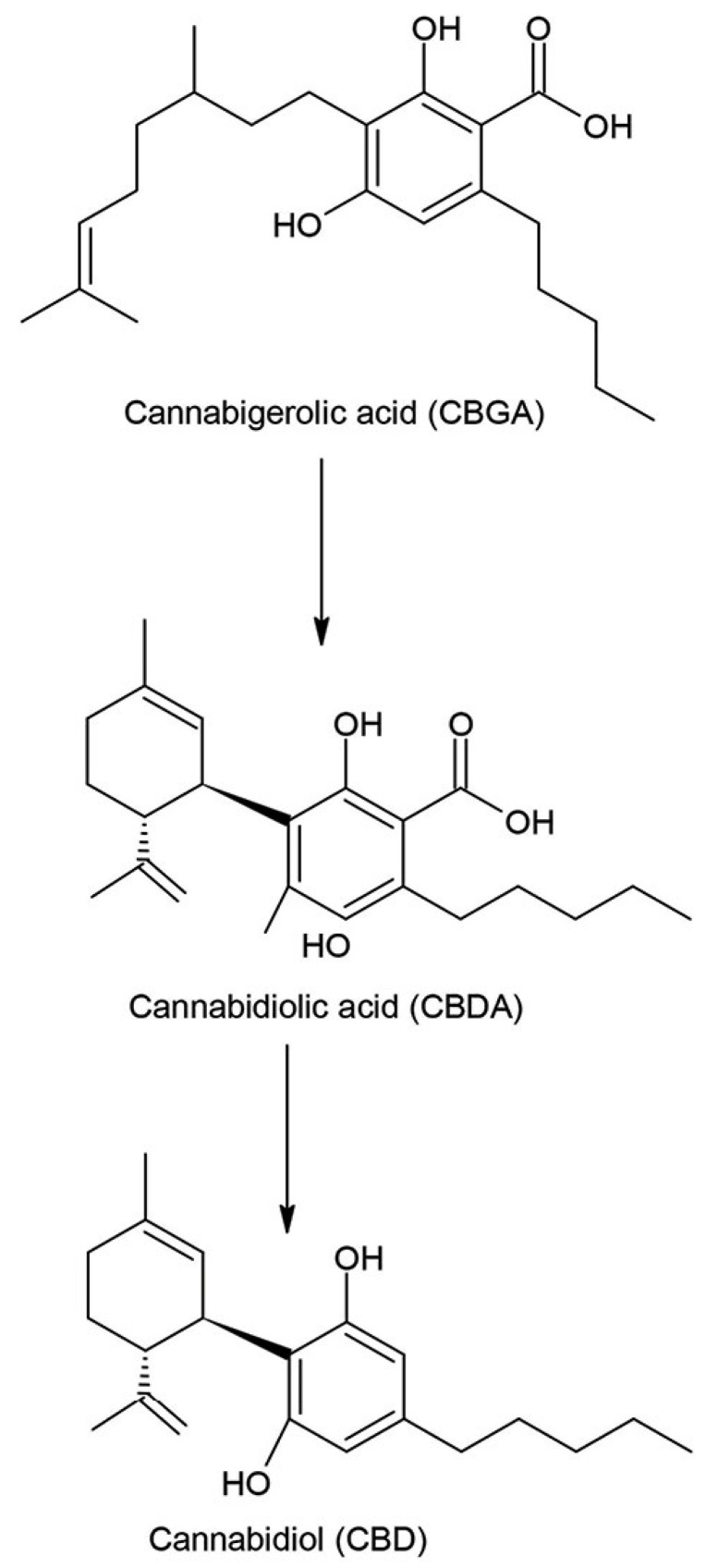
Hemp plants convert cannabigerolic acid (CBGA) into cannabidiolic acid (CBDA), which is then transformed in the laboratory to cannabidiol (CBD) and carbon dioxide (CO_2_) [3].

Cannabidiol (CBD) is one of at least 85 active cannabinoids identified within the Cannabis plant. Cannabidiol is a phytocannabinoid derived from Cannabis species, which is devoid of psychoactive activity, with analgesic, anti-inflammatory, antineoplastic and chemopreventive activities. Figure 4 illustrates the conversion of cannabigerolic acid (CBGA) into cannabidiol acid (CBDA) that takes place in hemp plants and the next transformation to cannabidiol (CBD) in the laboratory.

**Figure 5:**
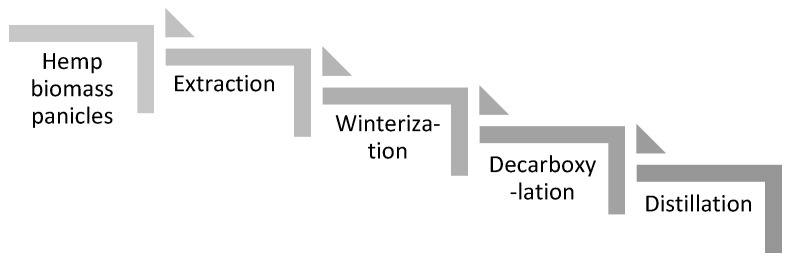
Scheme of hemp biomass conversion into high purity distillate.

CBD is obtained from the top part of hemp, e.g., from plant panicles. Main processing steps to convert hemp biomass into high purity distillate covers trim and flowers collection, extraction with crude oil, removing of fats by winterization, activation with decarboxylation, and purification by distillation, the chain is schematically showed in Figure 5. 

Many researchers have studied CBD’s effect on human health, several research papers report the benefits of CBD for the skin, including combating inflammation [4,5,6], pain [7], and melanoma; acting as an analgesic to promote wound healing [8].

In human trials, Palmieri et al. found that topical application of CBD improved skin lesions in the course of psoriasis, e.g., reduction of Psoriasis Area Severity Index, atopic eczema, and tissue scarring, as well as increased skin hydration and elasticity [9]. Likewise, Wilkinson showed that CBD acts on psoriasis in cultured human epidermal keratinocytes [10]. 

The positive effect of CBD on the human body was approved in many scientific articles, which provided high quality evidence for the neurological effect of cannabidiol in people [11,12,13,14]. 

However, in 2019, the European Commission announced that CBD and other cannabinoids were classified as “novel foods”, and products containing CBD must be approved as a novel food by a national food authority [15,16]. In December 2020, the European Commission concluded that CBD should not be considered a drug and can be qualified as food [17].

From an EU law perspective, CBD may be used in cosmetics placed on the EU market when obtained from cannabis, cannabis resin, cannabis extracts, and cannabis tinctures originating from the seeds and leaves that are not accompanied by the fruiting tops of the cannabis plant. Cannabidiol is listed in the EU Cosmetics Ingredient Database [18]. Regulation (EU) No 1223/2009 (the Cosmetics Regulation) defines a cosmetic product as any substance or mixture intended to be placed in contact with external parts of the human body for cleaning, perfuming, changing its appearance, protecting, keeping in good condition or correcting body odors [19]. 

Growing usage of cannabis for medical and recreational purposes, coupled with the holistic benefits of CBD-infused skincare products, is the key growth enabler for the market. The beauty and personal care industry is observing an alteration towards CBD-infused skincare products [20]. These product lines comprise hand care, lip moisturizer, and skincare products. 

**Figure 6:**
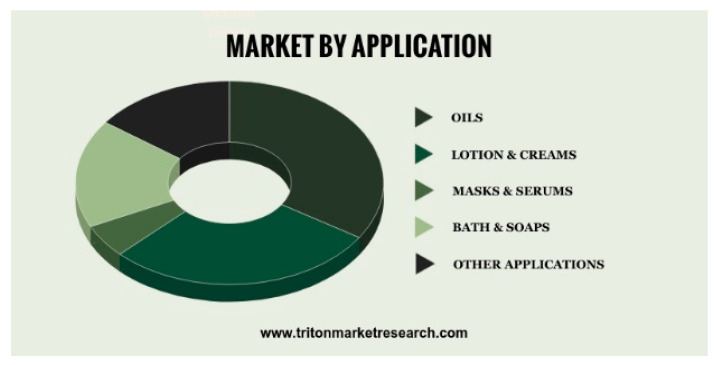
Segmentation of CBD application in the cosmetic sector [20].

The global CBD skincare market was valued at USD 551.57 million in 2019 and is expected to reach USD 3168.44 million by 2028, growing at a CAGR of 21.44% during the forecast period, Figure 6 [20]. However, so far, there is a lack of data about the use of cannabidiol for functional textile purposes.

The positive effect of CBD on human skin has been proved by many researchers. Palmieri et al. showed based on skin evaluations (hydration, TEWL, elasticity), clinical questionnaires, and supported by photographic data and investigators’ clinical assessment that topical treatment with CBD-enriched ointment significantly improved the skin parameters, the symptoms, and also the PASI index score. No irritant or allergic reactions were documented during the period of treatment. The topical administration of CBD ointment, without any THC, is a safe and effective non-invasive alternative for improving the quality of life in patients with some skin disorders, especially on inflammatory backgrounds [9]. Results of research conducted by A. Olah et al. proved that CBD had universal sebostatic activity, which was accompanied by substantial anti-inflammatory effects on human skin and would be very much desired in the clinical treatment of acne vulgaris [5]. CBD normalized the pathologically elevated lipogenesis induced by “pro-acne” agents, both quantitatively and qualitatively, suppressed cell proliferation, and prevented the actions of TLR activation or “pro-acne” agents to elevate proinflammatory cytokine levels (universal anti-inflammatory effect). 

CBD was shown to inhibit the proliferation of hyperproliferative keratinocytes [10] and it was demonstrated to possess remarkable antibacterial activity [21].

Cannabinoids have been found to have antioxidant properties, unrelated to NMDA receptor antagonism. This newfound property makes cannabinoids useful in the treatment and prophylaxis of a wide variety of oxidation-associated diseases, such as ischemic, age-related, inflammatory, and autoimmune diseases [22]. 

Knowing the bioactivity of hemp fibers and the influence of cannabidiol CBD extracted from hemp plants on human skin, the goal of this study was defined. The aim was to design of hemp fiber value chain to manufacture functional clothing, using hemp inherent biological activity reinforced by cannabidiol (CBD) applied on the fabric surface. To confirm the developed pure hemp fiber/CBD clothing effect on human skin, human wearing trials were conducted. The functional clothing made of hemp fibers and hemp CBD extract is an innovative solution with a high impact on both human health and wellbeing as well as environmental preservation. The use of cannabinoids to improve the biological activity of hemp textiles has not been reported in any scientific articles published so far. In literature, it is possible to find consideration about functional clothing, including cosmetotextiles improving skin moisture or showing antibacterial, antiaging deodorant and other activity, where the function is given by use of microcapsules or special finishing processes [23,24,25], but there is no data about functional hemp clothing enriched by CBD extract. This invention has been applied for a patent, No: P.438388, 2021.

## 2. Results and Discussion

The objective of the study was to develop bioactive clothing with the use of only pure hemp components, e.g., hemp fibers modified by Cannabidiol (CBD) extracted from industrial hemp panicles, and to assess its effect on the human skin. 

The textile raw material, e.g., hemp fibers extracted from the Bialobrzeskie variety after dew retting was evaluated in terms of fiber parameters and chemical composition. Characteristics of the fibers are presented in Table 1 and Table 2.

The technological parameters of hemp fibers proved their suitability for use in a flax spinning system for textile product purposes. The linear density at the level 0.9 tex means that technical bundles were dividing efficiently on smaller fiber complexes, thanks to applied processes: dew retting and mechanical scutching. 

Hemp fibers contained lignin, cellulose, hemicellulose, pectin, waxes, and fats in their chemical composition, Table 2. The value of cellulose content, 68.4% is lower in comparison to flax (71%) and significantly lower in comparison to cotton, where cellulose is about 98% [26,27]. Cellulose and hemicellulose affect the softness of the fibers making the technological processes easier, fibers can be easily straightened out and parallelly laid in fibers stream to form slivers, roving, and then yarn. Pectin glutted elementary fibers into bundles in the plant stem, but most of it was removed during the retting and scutching process. Waxes and fats content affects fiber friction ratio, its low value means the necessity to apply surface natural fats and waxes on the fiber during the technological processes. Lignin content influences fiber stiffness and makes difficulties in the spinning processes. On the other side, lignin is associated with phenolic acids in plants, in the crosslinking between lignin and hemicellulose in the cell wall carbohydrates [28,29,30,31]. Lignin content is correlated with active phenolic acids content in the bast fiber [2].

In the current study, the hemp fibers were extracted from the stem with the use of the dew retting process, where naturally occurring fungi affected by moisture and ambient temperature conditions were responsible for the disintegration of the fibrous plant stem to release fibers [32,33]. The enzymes secreted by fungi can support the antibacterial activity of dew retted fibers. This phenomenon is very limited in the case of the water-retting process because in that case activity of anaerobic bacteria is mainly responsible for fiber release. For this reason, the dew retting was selected to apply in this experiment.

Obtained long hemp fiber with linear density 0.9 tex allowed for the production of yarn with linear mass 85 tex produced with the use of the wet spinning system. The yarn parameters are presented in Table 3. The number of twists and mechanical properties of the hemp yarn were suitable for the weaving process. 

### 2.1. Hemp Fabric and Modification with CBD Extract

Two types of hemp fabric were produced: one with plain and the second with twill weave to give small diversification of raw materials for clothing designers. Twill fabric was characterized with bigger softness, higher air permeability, higher thermal and water vapor resistance in comparison to plain fabric, which resulted from threads setting in fabric structure. 

Characteristics of manufactured fabric are presented in Table 4. 

Results of hemp fabric parameters tests like hygroscopicity, ability to water sorption, air permeability, thermal and water vapor resistance indicated, that clothing made of this fabric will guarantee the comfort of wearing in the condition of everyday life. Additionally, the value of ultraviolet protection factor (UPF) means, that the hemp clothing will protect the user against UV radiation [34]. On the other side, mechanical parameters indicated good resistance to breakage and abrasion, which allowed predicting a long time of clothing use. The ability to use the clothing for a long time results as a consequence in limitation of creation of waste and is one of the tools of waste management necessary for environment protection. Value of bedding stiffness and angle recovery indicated the tendency of the fabric to crease, these features are typical for bast fiber woven materials. 

The pure hemp fabric was evaluated in terms of phenolic acids content, the results are presented in Table 5. The phenolic compounds are listed in the literature as antibacterial and antioxidant agents occurring in many different plants [35,36]. 

In the current study, the results of the HPLC tests confirmed the presence of *p*-coumaric, syringic, and ferulic acid in the chemical composition of the hemp fibers in the fabric. It is well established that the presence of syringic acid is correlated with high antioxidant and antibacterial activity [37]. *P*-Coumaric acid is mainly a plant metabolite that exhibits antioxidant and anti-inflammatory properties. Its bactericidal activity mechanism consists in damaging bacterial cell membrane and interacting with bacterial DNA [38,39]. Ferulic acid is a component of a primary cell wall and is bonded with lignin and hemicellulose in fibrous plants [40]. Strong antioxidant properties of the ferulic acid cause that is used, apart from other applications, as a cosmetic ingredient to protect the skin against unfavorable effects of the environment and to act as an antiaging agent [41,42]. The proven phenolic acids present in the hemp fiber structure resulted in the bioactivity of developed fabric, particularly its antioxidant properties.

The values of the FRAP tests shown in Table 6 confirmed the antioxidant activity of the hemp fabric, which is in line with earlier studies and findings available in the scientific literature [2]. 

The developed pure hemp fabric having direct contact with the skin of the clothing wearer will protect the human skin against harmful factors of the environment like free radicals or UV rays thanks to fiber antioxidant activity. The fabric was safe for users, the test of cytotoxicity proved not to be cytotoxic to keratinocytes as it did not influence its proliferation in the cytotoxic examination. Tested material showed slight antibiotic activity (Table 7), in the case of pure hemp fabric, it is a favorable feature for humans in their everyday life and this is added value of the functional clothing. 

**Figure 7:**
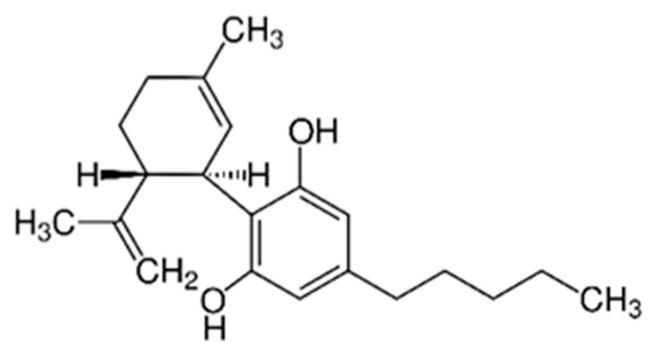
CBD structure.

To strengthen the fabric bioactivity and to build a bigger potential of clothing to act as cosmetics, extracts from panicles of *Cannabis sativa* L., e.g., cannabidiol were applied on the material surface. CBD extract in the form of microcapsules was addressed to the textile target. The chemical structure of CBD is shown in Figure 7.

Microcapsules with CBD extract were commercially purchased from Devan company. In the experiment, the microcapsules were applied on the fabric surface with the use of a suspension concentration of 5 g/m² by the padding method. The microcapsules were bonded to the hemp fabric surface by use of a mix of agents, including a cross-linker specially developed for the thermic process to permit the covalent bonding of microcapsules directly to textile fibers. 

**Figure 8:**
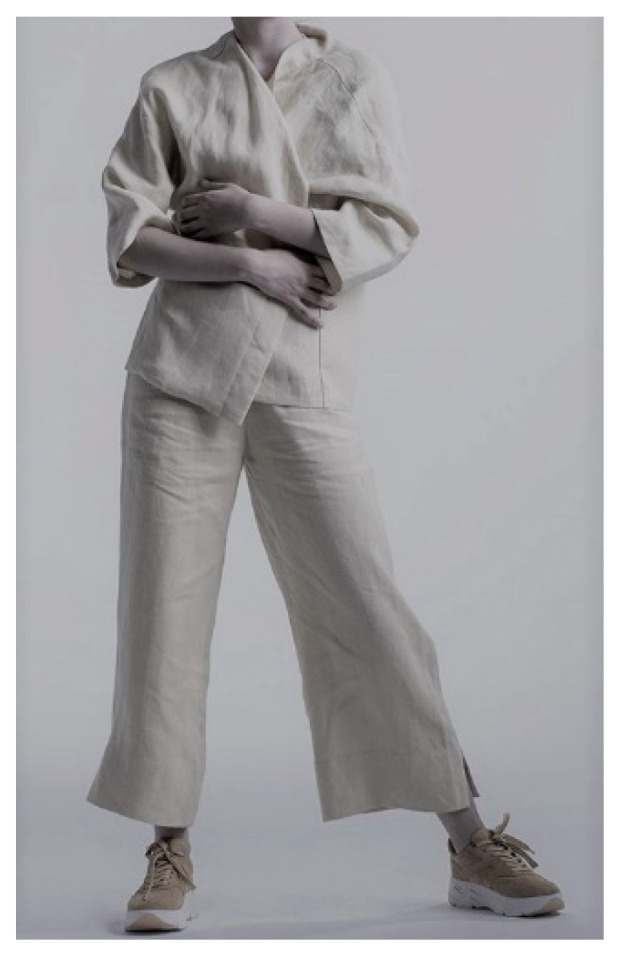
Hemp clothing functionalized by CBD extract.

The clothing was design to ensure direct continuous contact of active fabric surface with the largest part of human skin, e.g., with long sleeves and long trousers. This guarantees that Cannabidiol presents on the fabric surface is absorbed by skin-supporting neutralization of reactive oxygen spaces and acting as body cosmetic. The example of functional clothing is shown in Figure 8. 

The microcapsules with CBD were applied to the fabric to give hemp clothing stronger functionality in terms of skincare. 

The hemp clothing with microcapsules on its surface was evaluated in terms of the wearing and washing fastness. The test results indicated, that after 6 weeks of clothing use and 20 cycles of washing with the application of gentle process without detergents, most of the microcapsules were present on the fabric surface.

### 2.2. The Human Trials

The human trials were conducted with the participation of 15 volunteers wearing tested clothing 12 h/day for six weeks. The skin parameters were tested before and after the experiment and the results of the tests are presented in Table 8, Table 9 and Table 10. 

As shown in Table 8, TEWL (mean by 2.487 g/m^2^/h) statistically significantly decreased, and skin hydration measured by corneometry (mean by 10.856 AU) increased statistically significantly after 6 weeks of wearing the clothing. Both of these biophysical parameters reflect the barrier function of the epidermis. TEWL refers to the loss of water in the form of water vapor through the epidermis by passive diffusion without the participation of sweat glands and its decrease is beneficial for skin barrier function The epidermal water content is assessed by measuring the electrical capacitance The water content of the epidermis is assessed by measuring the electrical conductivity or electrical resistance of the skin. which, due to the dielectric constant of water, change with the degree of hydration of the epidermis, making it possible to evaluate the epidermal barrier. The skin hydration value increased after a period of wear test. In all subjects, the epidermal barrier function was normal both before and after the study. Skin pH and sebum secretion did not change. There was a statistically significant decrease in skin gloss (mean by 3.513 GU). Melanin index (MI) and erythema index (EI) indicate an increase in hemoglobin content with no difference in melanin content.

Table 9 presents the results of subjective assessment of the properties of wearing clothing. Most volunteers positively assessed the comfort of wearing, softness, and convenience of textile. None of the subjects experienced redness of the skin, 4 women noticed a difference in skin hydration. Almost half of the respondents positively determined the durability of the aesthetic properties of the product.

Table 10 shows the results of the feelings related to the wearing of the tested clothing according to the NRS scale. The majority of respondents rated clothing as pleasant in contact with the skin, thermally neutral, not irritating, and not affecting the smoothness of the skin.

TEWL is a noninvasive in vivo measurement of water loss across the stratum corneum [43,44].

Skin surface hydration could be assessed with another noninvasive method called corenometry. The disrupted epidermal barrier causes the skin to be susceptible to irritants, allergens, and microbes. Textiles are one of the environmental factors with potential irritants or allergic properties. During the aging process, the skin barrier becomes impaired. Textile that helps to restore its parameters may be useful in the protection of the skin from damaging environmental factors. 

The results of the current study are in line with research conducted by Palmieri et al. [9] on CBD effect on the skin. Palmieri proved significant improvement of skin parameters after three months of hemp touch organic skincare ointment contained CBD seed oil use by 20 volunteers. After a treatment period, the tested parameters of human skin like hydration, TEWL, and elasticity were significantly improved in all the patients. 

The results of the biophysical examinations of volunteers wearing hemp clothing enriched with CBD extracts for six weeks, showed increasing hydration of the stratum corneum of the skin as well as a decrease in TEWL, which may indicate the improvement of skin barrier function. Subjective patient opinions about the wearing of the developed clothing were positive. 

## 3. Material and Methods

### 3.1. Hemp Fiber, Yarn, and Fabric Preparation

Hemp variety, Bialobrzeskie, for fibers purpose was cultivated under controlled conditions without the use of pesticides in the Experimental Station of IWNiRZ PIB in 2018. After dew retting on the field for 7 weeks, the straw was conditioned under the roof in ambient conditions during the winter season. The dew retting of hemp straw was applied to ensure high bioactivity of fibers according to results of the research described by Zimniewska et al. [2]. The fiber extraction from the dew retted straw to obtain long fibers was conducted with the use of scutching turbines. Hemp yarn produced by the flax spinning system was used as weft and warp for the weaving process. Softening of hemp fabric was conducted only with the use of mechanical processes without silicones application to reduce chemical use.

### 3.2. Assessment of Fiber, Yarn, and Fabric Parameters

The parameters and chemical composition of fiber extracted from hemp straw variety, Bialobrzeskie, were evaluated based on relevant international and national standards.

List of Standards used for fiber tests:

- Linear density of the fibers: PN-EN ISO 1973:2011
- Length of the fibers: PN-ISO 6989:2000
- Indices (breaking force, elongation, and tenacity) at static tension: PN-P-04676:1986

List of Standards used for yarn tests:

- Linear density of the yarn: PN-EN ISO 2060:1997
- Twist number: PN-EN ISO 2061:2015
- Single-end breaking force, elongation at break, and tenacity using constant rate of extension tester: PN-EN ISO 2062:2010

List of Standards used for fabric tests:

- Mass per square meter: PN-ISO 3801:1993
- Thread density: PN-EN 1049-2:2000
- Hygroscopicity at relative humidity of air 65% and 100%: PN-P- 04635:1980
- Water sorption (drop method): JIS 1090:1990
- Air permeability: PN-EN ISO 9237:1998
- Thermal resistance and water-vapor resistance under steady-state conditions (sweating guarded-hotplate test): PN-EN ISO 11092:2014-11
- Solar UV protective properties; Method of test for apparel fabrics: PN-EN 13758-1 + A1:2007
- Maximum force and elongation at maximum force using the strip method: PN-EN ISO 13934-1:2013-07
- Bending stiffness: PN-EN ISO 9073-7:2011
- Angle of recovery: PN-EN 22313:2000
- Abrasion resistance: PN-EN ISO 12947-2:2017-02

The chemical composition of the hemp fibers was evaluated with the use of relevant standards:

- Waxes and fats content: Branch Standard BN-86/7501-10
- Pectin content: a method developed at INF&MP
- Lignin content: Branch Standard BN-86/7501-11
- Cellulose content: PN-92/P-50092
- Hemicelluloses content: Branch Standard BN-77/7529-02
- Phenolic acids, e.g., coumaric and ferulic acid, in hemp fibers were assessed by the high-performance liquid chromatography (HPLC) method.

Additionally, lignin extracted from dew retted hemp fibers were tested to show the presence of phenols. Lignin was extracted from fibers according to the method described in the standard: BN-86/7501-11. The Fourier Transform Infrared Spectroscopy was used to determine absorption spectra for lignin. The trials were done with a Spectrophotometer FT-IR NICOLET iS10, (Thermo Scientific, Waltham, MA, USA) at infrared wavelengths of 350–4000 cm^−1^.

### 3.3. Assessment of Fiber Antioxidant Activity

Antioxidants are a large group of natural and synthetic compounds with the ability to reduce free radicals and prevent some amount of the oxidative damage that destroys and depletes the skin function and structure while also preventing some of the degenerative effects in the skin caused by sun exposure. In this study, the method of Ferric Ion Reducing Antioxidant Parameter (FRAP) was used for testing hemp fibers’ antioxidant capacity [45].

**Figure 9:**
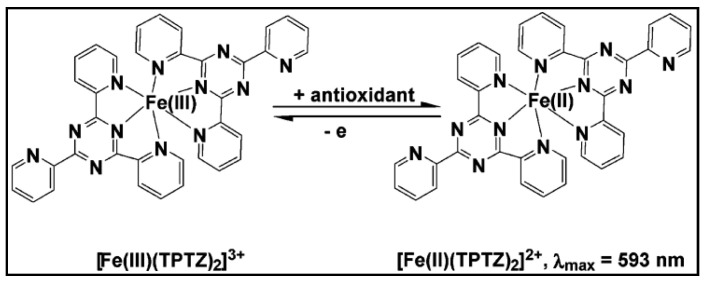
FRAP—electron-transfer reaction.

The Ferric Ion Reducing Antioxidant Power (FRAP) assay takes advantage of electron-transfer reactions, Figure 9. The ferric reducing activity (FRAP) of the fibers extracts was estimated according to the method developed by Benzie and Strain [46]. The reaction mixture contained 300 mmol/L acetate buffer, 10 mmol/L 2,4,6-tripyridyl-s-triazine (TPTZ) in 40 mmol/L of HCL and 20 mmol/L of FeCl3·6H2O. The working FRAP reagent was prepared freshly by mixing 25 mL of acetate buffer, 2.5 mL of TPTZ solution, and 2.5 mL of FeCl3·6H2O. The freshly prepared mixture was incubated at 37 °C in a water bath for five minutes and then a blank reading was taken spectrophotometrically at 593 nm. After that, 30 µL of extract or standard and 90 µL of distilled water were added to 900 µL of the working FRAP reagent. Absorbance was measured at 0 min immediately upon addition of the working FRAP reagent after vortexing. Thereafter, absorbance reading was taken after four minutes [36,47]. Three repetitions of the antioxidant test were conducted for each fiber sample.

Antibiotic activity test method: 20 samples of fabric were used for the test. The liquid extracts were prepared with the use of a bacterial base (CASO Broth, Merck) (1210C, 20 min). 1 mL of fabric extract was added to the standard strain of S. aureus—ATCC 6538 [48]. Incubation for 18 h, temp. 37 °C. Determination of minimal concentration of fiber extracts which inhibit standard strain of S. aureus growing (MIC-Minimal Inhibitory Concentration) [49,50]. 

Cytotoxic studies were conducted on human keratinocytes (Human Epidermal Keratinocytes, adult) grown in medium culture Medium EpiLife^®^ supplemented with growth factors (Human Keratinocyte Growth Supplement) and antibiotics (gentamicin and amphotericin) [51]. 

### 3.4. Human Wearing Trial

The clinical trial was conducted in a group of randomly recruited women who gave their informed consent and agreed to wear the hemp clothing for 6 weeks for 12 h per day. The study included an assessment of the dermatological status and measurements of skin biophysical parameters in 15 volunteers aged 52 to 79 years (mean 57.8 ± 4.6). Before the wearing test, a size-matched hem clothing was prepared for each participant. None of the volunteers reported skin diseases, and the skin condition was described as normal. The experiment of wearing clothes was conducted in 6 summer weeks. Skin examination was performed twice: before the wear test and after 6 weeks of the usage of hemp clothing. Corneometry, transepidermal water loss (TEWL), sebumetry, and skin pH were measured with noninvasive methods using the MPA 9 System (Courage-Khazaka) [52]. The pigmentation and erythema were examined using the reflection spectrophotometer (Mexameter MX18^®^ Courage + Khazake electronic GmbH; Köln, Germany) and presented as melanin index (MI) and erythema index (EI) in Arbitrary Units (AU) [53]. Lower absorption of light measuring the level of melanin in the area of hyperpigmentation indicates skin lightening, lower absorption of the light measuring the level of hemoglobin indicates a reduction in erythema. Glossymeter GL200 expresses the portion of directly reflected light (gloss) and the diffusely scattered portion from the skin surface. The measurement of gloss is based on the reflection of light sent to the skin (Courage + Khazaka electronic GmbH). The measurements were performed under the same ambient conditions: constant relative humidity of about 40% and temperature of 20 °C, after 10–15 min acclimatization of the subjects to stabilize blood circulation. 

All participants evaluated the clothes after 6 weeks of use, according to the observation card. The answers “yes” or “no” concerned: the comfort of wearing, the softness of the product, the occurrence of redness of the skin during the use of clothing, noticeable changes in skin hydration, the durability of aesthetic properties, and ease of maintenance (washing) of clothing. To assess the subjective impact of clothing on the skin, a numeric rating scale (NRS) from “0” to “10” was used, where 0 meant the shoulder of satisfaction and 10 was the maximum satisfaction. The following were evaluated: sensations in contact with the skin (0 meant unpleasant sensations and 10 very pleasant), the thermal effect of wearing clothing (0 meant cooling, 5 neutral, and 10 warming), the effect of clothing on the skin (0 meant irritating, 5 neutral, and 10 soothing), skin smoothness (0 meant the occurrence of skin roughness, 5 no changes, and 10 pronounced smoothness), material from which the clothing was made (0 meant skin-unfriendly material, 5 neutral and 10 skin-friendly), comfort of use (0 means lack of comfort, 5 neutral, and 10 high comfort of use). 

The study was approved by the Bioethics Committee of the Poznan University of Medical Sciences (352/20, 14 May 2020). Informed written consent was obtained from all participants.

Microsoft Excel v. 16.37 (Redmond, WA, USA), and Statistica 14 (StatSoft) (Palo Alto, Santa Clara, CA, USA) were used for statistical analysis. Data are presented as means, and standard deviations (SD), minimum and maximum values, or percentage, as appropriate. The Wilcoxon rank test was used to compare the difference in examined parameters before and after the clothing ware. The *p*-value of 0 < 0.05 was considered statistically significant.

## 4. Conclusions

Results of the study proved the possibility to produce advanced hemp textiles, where only natural hemp components have been used. 

Application of microcapsules to hemp fabric ensures the functionality of clothing resulted from hybrid bioactivity created by combining two active pure hemp components: CBD and fibers processed according to strong technological restrictions. 

The developed clothing improves the hydration and lubrication of the skin, helps to keep the proper moisture and gloss of the skin, and enhancing the skin barrier function. The hemp fabric used for the clothing proved to be safe and comfortable, nor irritant or allergen for the skin. The hemp clothing enriched by Cannabidiol shows a high impact not only on the human skin but also on the environment.

The presented solution indicated a new possible direction of clothing technologies development, which are in agreement with principles of The European Green Deal strategy. Production of raw materials, beginning from hemp cultivation allows keeping biodiversity in agriculture, which is important for the protection of the ecosystem, reduction of water and pesticides consumption if compared to cotton, absorbing CO_2_ from the atmosphere, and supports a wasteless bioeconomy. 

## Figures and Tables

**Table 1:** Hemp fiber parameters.

Fiber Parameter	Unit	Mean/Standard Deviation (SD)	Test Result
Linear density	tex	Mean	0.9
		SD	0.17
Length	mm	Mean	454
		SD	-
Breaking force	N	Mean	46.65
		SD	7.25
Elongation	%	Mean	36.09
		SD	3.18
Tenacity	cN/tex	Mean	22.09
		SD	2.61

**Table 2:** Chemical composition of hemp long fibers.

Lignin Content	SD	Cellulose Content	SD	Waxes and Fats Content	SD	Hemicellulose Content	SD	Pectin Content	SD
(%)	(%)	(%)	(%)	(%)	(%)	(%)	(%)	(%)	(%)
7.65	0.10	68.40	0.93	1.04	0.31	20.16	0.05	1.66	0.15

**Table 3:** The hemp yarn parameters.

Yarn Parameters	Unit	Mean/Standard Deviation (SD)	Test Result
Linear density	tex	Mean	85
		SD	0.2
Twist number	T/m	Mean	262
		SD	8.37
Breaking force	N	Mean	20.01
		SD	4.54
Elongation	%	Mean	1.96
		SD	0.28
Tenacity	cN/tex	Mean	24.11
		SD	5.47

**Table 4:** Properties of pure hemp fabrics.

	Fabric Parameters		Unit	Mean/Standard Deviation (SD)	Plain Weave Fabric	Twill Weave Fabric
Fabric structure	Mass per square meter		g/m^2^	Mean	267	271
				SD	0.6	3.4
	Threads density l/dm	warp	-	Mean	153	155
				SD	0.5	0.7
		weft		Mean	141	144
				SD	0.5	0.5
Comfort parameters	Hygroscopicity	65% relative humidity of air	%	Mean	7.70	7.92
				SD	0.22	0.13
		100% relative humidity of air		Mean	12.78	12.81
				SD	0.39	0.14
	Water sorption		s	Mean	9	4
				SD	1.8	0.6
	Air permeability		mm/s	Mean	607	822
				SD	23	21
	Thermal resistance		m^2^ C/w	Mean	0.0307	0.0527
				SD	0.0003	0.0009
	Water vapour resistance		m^2^ Pa/W	Mean	5.5765	10.6560
				SD	0.1865	0.0257
	UPF		-	Mean	35	25
Mechanical properties	Breaking force	warp	N	Mean	748.48	779.66
				SD	33.98	22.81
		weft		Mean	871.50	762.58
				SD	45.82	41.00
	Elongation in breaking force	warp	%	Mean	16.81	7.68
				SD	0.90	0.59
		weft		Mean	14,07	14,84
				SD	0.31	0.29
	Bending stiffness	warp	m N cm	Mean	15.48	21.50
				SD	1.20	1.33
		weft		Mean	14.14	9.11
				SD	1.29	0.82
	Angle of recovery	Warp right side	^O^	Mean	71	74
				SD	5.3	5.4
		Warp left side		Mean	75	76
				SD	6.0	6.2
		Weft right side		Mean	68	82
				SD	8.8	5.7
		Weft left side		Mean	83	88
				SD	5.0	7.3
	Abrasion resistance		number of cycles		18,000	10,000

**Table 5:** Phenolic acids content in a pure hemp fabric.

Content of Acids in Hemp Fabric:					
Syringic (mg/100 g)		*p*-Coumaric (mg/100 g)		Ferulic (mg/100 g)	
Result	SD	Result	SD	Result	SD
0.178	0.008	0.013	0.000	0.645	0.007

**Table 6:** Antioxidant activity of pure hemp fabric assessed by the FRAP method.

	Sample Concentration (mg/mL)	FRAP (μmol/L)	Mean FRAP (μmol/L)	SD
Hemp fabric	0.378	22.87	23.04	0.17
		23.04		
		23.21		
	0.756	41.24	39.59	2.91
		41.34		
		36.21		
	1.513	83.40	73.99	11.62
		77.58		
		61.00		
	2.269	120.33	111.21	7.93
		105.97		
		107.34		
	3.025	151.45	149.80	14.86
		134.18		
		163.76		

**Table 7:** Bioactivity, e.g., antibiotic units (JA), minimal inhibitory concentration (MIC), and cytotoxicity of hemp fabric.

Cytotoxicity (%)	MIC (mg/mL)	JA/g
No toxic effect	240	4.2

**Table 8:** Comparison of the skin biophysical parameters before and after 6 weeks of using the tested clothing.

Type of Test	Results			*p*-Value
	Before	After	Difference	
	M ± SD	M ± SD	M ± SD	
TEWL (g/m^2^/h)	10.007 ± 4.674	7.520 ± 1.283	2.487 ± 5.139	0.020
Corneometry (AU)	44.370 ± 8.717	56.214 ± 10.856	11.844 ± 8.784	0.000
Sebumetry (AU)	1.533 ± 2.167	2.433 ± 3.396	0.900 ± 3.790	0.373
pH	4.842 ± 0.775	5.144 ± 0.810	0.302 ± 0.949	0.238
Glossymetry (GU)	27.913 ± 4.586	31.427 ± 4.167	3.513 ± 3.310	0.001
MI (AU)	112.801 ± 39.624	120.245 ± 55.940	7.445 ± 22.270	0.216
EI (AU)	217.555 ± 59.211	241.028 ± 81.475	23.473 ± 41.547	0.046

Blue color: statistically significant difference. M: mean, SD: standard deviation, AU: arbitrary unit, GU: glossary unit, MI: melanin index, EI: erythema index.

**Table 9:** Subjective assessment of the properties of clothing during use.

Characteristic	Results	
	Yes *n* (%)	No *n* (%)
Comfortable wearing	13 (87)	2 (13)
Softness of the product	12 (80)	3 (20)
Redness of the skin when worn	0	15
Noticeable changes in skin hydration	4 (73)	11 (27)
Durability of aesthetic properties	8 (53)	7 (47)
Ease of maintenance	13 (87)	2 (13)

**Table 10:** Subjective assessment of sensations during the 6 weeks of use of clothing.

Parameters	Results (Points) M ± SD	Range (Points) Min–Max
Sensations in contact with the skin	8.5 ± 1.4	6–10
Thermal effect of wearing clothing	6.1 ± 2.1	2–10
Effects of clothing on the skin	5.8 ± 1.9	4–10
Subjective assessment of skin smoothness	5.9 ± 1.7	4–10
Subjective assessment of the material from which the clothing was made	7.5 ± 2.3	4–10
Assessment of the comfort of use	6.1 ± 3.1	0–10

M: mean, SD: standard deviation.

## Data Availability

The data presented in this study are available on request from the corresponding author. The data are not publicly available due to protection of patent application.
